# Complete chloroplast genome of ornamental orchids *Eulophia flava*

**DOI:** 10.1080/23802359.2019.1664946

**Published:** 2019-09-13

**Authors:** Haili Li, Renmao Li, Jianmin Zhang, Qianting Yin, Ying Zhang

**Affiliations:** Life Science and Technology School, Lingnan Normal University, Zhanjiang, China

**Keywords:** *Eulophia flava*, ornamental orchids, chloroplast genome, phylogenomic tree

## Abstract

*Eulophia flava* (Lindl.) Hook.f. (Orchidaceae) is a high value ornamental plant especially for cutting flowers in China. In this paper, we reported and characterized the complete chloroplast genome sequence of *E. flava* by assembling from short reads generated by Illumina sequencing. Total chloroplast genome size was 148,903 bp, including inverted repeats (IRs) of 26,651 bp, separated by a large single copy (LSC) and a small single copy (SSC) of 84,465 and 13,132 bp, respectively. A total of 119 genes, including 36 tRNA, 8 rRNA, and 75 protein-coding genes, were identified. The GC content of *E. flava* is 36.8%. Phylogenetic analysis using the maximum-likelihood algorithm showed that *E. flava* is sister to a clade with one species in the genus *Eulophia*.

*Eulophia flava* (Lindl.) Hook.f. (Orchidaceae) is a high-value ornamental plant, especially for cutting flowers. It is mainly distributed not only in south of Guangdong province and Guangxi province, Hainan province, and Hong Kong in China but also in Nepal, India, Myanmar, Vietnam, and Thailand (Wang and Chen 2001). Now, researches on *E. flava* have focused on its growth and development (Yin et al. 2010; Zhang et al. 2010; Wang et al. 2015). The chloroplast (cp) DNA carries rich information for plant molecular systematics and has been widely used to understand plant genetic diversity . However, there have been no reports on cp genome information of *E. flava* until now. In this study, the complete cp genome of *E. flava* was characterized to provide the underlying information for genetic breeding and conservation studies of this species.

Fresh leaves were collected from one individual of *E. flava* in Qishui Town, Leizhou City (N 20°43′, E 109°50′), China. The total genomic DNA was extracted from five mixed fresh leaves of *E. flava* by using the modified CTAB method (Doyle 1987) in the laboratory of Lingnan Normal University (Accession number: LNH180603035). Further, the specimen was also stored in the herbarium of Lingnan Normal University. Genome sequencing was performed on an Illumina Hiseq X Ten platform with paired-end reads of 300 bp. In total, 48.8 Mb short sequence data with Q20 of 96.96% was obtained. The remaining high-quality reads were used to assemble the chloroplast genome in SOAPdenovo2 (Luo et al. 2012). The genes annotation in the chloroplast genome was done with the DOGMA Program with manual check and the circular chloroplast genome map was drawn by using OGDRAW (Wyman et al. 2004). The accession number in Genbank is MK855051. Eleven chloroplast genome sequences were aligned by using the complete chloroplast genomes, 10 plants from Orchidaceae including *E. flava*, and *Oryza sativa* were used as the out-group species. Phylogenetic analysis using the maximum-likelihood algorithm was conducted by using Mega X (Kumar et al. 2018).

A typical quadripartite structure was found in the chloroplast genome of *E. flava* with a length of 148,903 bp, which contains inverted repeats (IRs) of 26,651 bp, separated by a large single copy (LSC) and a small single copy (SSC) of 84,465 and 13,132 bp, respectively. The chloroplast genome has a GC content of 36.8% and contains a total of 121 predicted genes, including 36 tRNAs, 8 rRNAs, and 75 protein-coding genes. Among nine genes with intron, nine protein-coding genes (*rps*16, *atp*F, *rpo*C1, *pet*B, *pet*D, *rpl*16, *rpl*2, *ndh*A, and *ndh*B) contain one intron and two genes (*clp*P and *ycf3*) contain two introns. Phylogenetic analysis with other seven plant chloroplast genomes showed that *E. flava* is classed into a clade with another *Eulophia* species in Orchidaceae (Figure 1). The useful genomic resources for characterization of genetic diversity of *E. flava* by the chloroplast genome will help for the study of evolution mechanism in Orchidaceae.

**Figure 1.**
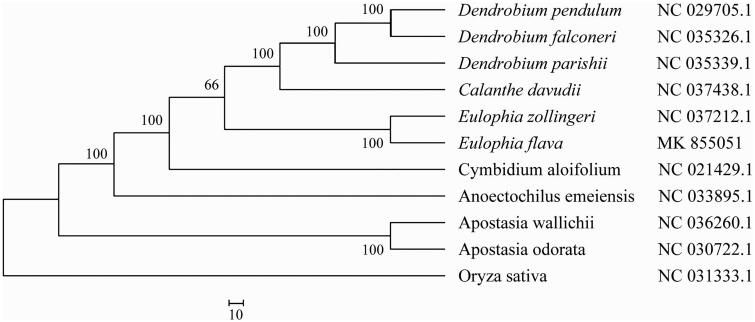
Maximum-likelihood tree based on the sequences of 11 complete chloroplast genomes. Numbers in the nodes were bootstrap values from 1000 replicates. Scale in substitutions per site.
